# Comparative analysis of basal and etoposide-induced alterations in gene expression by DNA-PKcs kinase activity

**DOI:** 10.3389/fgene.2024.1276365

**Published:** 2024-03-21

**Authors:** Sk Imran Ali, Mohammad J. Najaf-Panah, Kennedi B. Pyper, F. Ester Lujan, Johnny Sena, Amanda K. Ashley

**Affiliations:** ^1^ Department of Chemistry and Biochemistry, New Mexico State University, Las Cruces, NM, United States; ^2^ National Center for Genome Resources, Santa Fe, NM, United States

**Keywords:** DNA-PKcs, DNA damage repair, non-homologous end-joining, etoposide, gene expression, transcriptome

## Abstract

**Background:** Maintenance of the genome is essential for cell survival, and impairment of the DNA damage response is associated with multiple pathologies including cancer and neurological abnormalities. DNA-PKcs is a DNA repair protein and a core component of the classical nonhomologous end-joining pathway, but it also has roles in modulating gene expression and thus, the overall cellular response to DNA damage.

**Methods:** Using cells producing either wild-type (WT) or kinase-inactive (KR) DNA-PKcs, we assessed global alterations in gene expression in the absence or presence of DNA damage. We evaluated differential gene expression in untreated cells and observed differences in genes associated with cellular adhesion, cell cycle regulation, and inflammation-related pathways. Following exposure to etoposide, we compared how KR versus WT cells responded transcriptionally to DNA damage.

**Results:** Downregulated genes were mostly involved in protein, sugar, and nucleic acid biosynthesis pathways in both genotypes, but enriched biological pathways were divergent, again with KR cells manifesting a more robust inflammatory response compared to WT cells. To determine what major transcriptional regulators are controlling the differences in gene expression noted, we used pathway analysis and found that many master regulators of histone modifications, proinflammatory pathways, cell cycle regulation, Wnt/β-catenin signaling, and cellular development and differentiation were impacted by DNA-PKcs status. Finally, we have used qPCR to validate selected genes among the differentially regulated pathways to validate RNA sequence data.

**Conclusion:** Overall, our results indicate that DNA-PKcs, in a kinase-dependent fashion, decreases proinflammatory signaling following genotoxic insult. As multiple DNA-PK kinase inhibitors are in clinical trials as cancer therapeutics utilized in combination with DNA damaging agents, understanding the transcriptional response when DNA-PKcs cannot phosphorylate downstream targets will inform the overall patient response to combined treatment.

## Introduction

Eukaryotic cells frequently experience genotoxic stress from various exogenous and endogenous sources including exposure to UV radiation, reactive oxygen/nitrogen species formed during metabolism, and genotoxic chemicals, inducing myriad types of DNA damage (Lindahl and Barnes, 2000). DNA double-strand breaks (DSB) are deleterious and if left unrepaired or misrepaired can cause genomic instability associated with multiple diseases, including cancer. In response to DSB, cells initiate a complex, coordinated set of pathways, collectively known as DNA damage response (DDR), which involves damage sensing, transferring signals via signal transducers, and the response of many effector proteins to damage. Ideally, the DDR serves to repair DNA damage, when possible, through activation of repair proteins and upregulating transcription of additional genes needed for a full response. However, if repair is not feasible, the DDR initiates apoptotic death. In eukaryotes, the majority of DSB are repaired by the non-homologous end-joining (NHEJ) and homologous recombination (HR) pathways. The DNA-dependent protein kinase catalytic subunit (DNA-PKcs), a member of the phosphatidyl inositol 3-kinase-related kinase (PIKK) family of proteins, including ATM and ATR, is directly involved in NHEJ-mediated repair (Blackford and Jackson, 2017). DNA-PKcs is recruited to DSB by the Ku 70/80 heterodimer, forming the catalytically active DNA-PK holoenzyme, which in turn results in DNA-PKcs autophosphorylation and phosphorylation of other proteins, including Ku, H2AX, and RPA32. Additionally, DNA-PKcs serves to tether the two ends of the broken DNA (Yoo and Dynan, 1999) and activates other NHEJ proteins, XRCC4, XLF, Lig4 and PAXX, to facilitate DNA end-processing and ultimately ligation (Neal and Meek, 2011; Xing and Oksenych, 2019). DNA-PKcs regulates proteins involved in HR, including ATM, WRN, RPA32, cAbl and SMC1, facilitating crosstalk between the DDR proteins and two dominant DSB repair pathways (Karmakar et al., 2002; Collis et al., 2005; Shrivastav et al., 2008; Liu et al., 2012; Ashley et al., 2014; Blackford and Jackson, 2017). DNA-PKcs phosphorylates KAP-1 immediately after DSB and promotes chromatin decondensation to facilitate the recruitment of DDR proteins (Lu et al., 2019). Clearly, DNA-PKcs has multifaceted roles in regulating the cellular response to DSB.

DNA-PKcs also partakes in regulating proteins involved in cellular transcription. DNA-PKcs phosphorylates Snail1, a zinc-finger transcription factor involved in the epithelial-to-mesenchymal transition, which promotes its stability and function potentiation (Goodwin and Knudsen, 2014). DNA-PKcs is also involved in the activation of several metabolic genes. DNA-PKcs activation in the hypoxic condition protects HIF1α from degradation, which promotes the expression of GluT1, one of its target genes (Bouquet et al., 2011). DNA-PKcs promotes fatty acid biosynthesis in an insulin-dependent manner by activating the transcription factor USF-1 (Wong et al., 2009). During aging, DNA-PKcs phosphorylation of the heat-shock protein HSP90α decreases its mitochondrial function in skeletal muscles, thus decreasing overall metabolism and fitness (Park et al., 2017). DNA-PKcs was originally isolated as a component of the SP1 transcriptional complex as well as co-eluted with the largest subunit of RNA polymerase II (RNAPII) (Jackson et al., 1990; Maldonado et al., 1996). DNA-PKcs phosphorylates many transcriptional regulators, including RNAPII and RNAPI, TBP, TFIIB, TRIM28, Sp1, Oct1 and Oct2, AR, NRE, c-Myc, and c-Jun (Dvir et al., 1992; Lees-Miller, 1996). The availability of DNA-PKcs at the active transcription sites modulates the function of many transcription factors like autoimmune regulator (AIRE), p53, and erythroblast transformation-specific related gene (ERG) by direct interactions (Goodwin and Knudsen, 2014). DNA-PKcs may mediate transcription initiation by interacting with TopoIIβ and PARP1 in the presence of DSB (Ju et al., 2006; Ju and Rosenfeld, 2006). The effects of DNA-PKcs on global gene expression are not well-characterized. In this study, we analyzed global gene expression with and without DNA damage in cell lines expressing wild-type or kinase-inactive DNA-PKcs to elucidate the roles DNA-PKcs has in regulating gene expression in kinase-dependent and -independent manners, alone or following damage.

## Materials and methods

### Cell culture

V3-derived Chinese hamster ovary (CHO) cell lines were kindly provided by Dr. Katherine Meek, complemented with either human wild-type (WT) or kinase-inactive (K3753R (KR)) DNA-PKcs (Neal et al., 2014). Cells were cultured in α-MEM (Life Technologies, Waltham, MA) supplemented with 10% FBS (Millipore Sigma, St. Louis, MO), 1% penicillin/streptomycin (Life Technologies), 200 μg/mL G418 (Life Technologies), and 10 μg/mL puromycin (Santa Cruz Biotechnology, Santa Cruz, CA) at 37°C with 5% CO_2_ and 100% humidity. All chemicals were purchased from Millipore Sigma unless otherwise indicated.

### Immunoblotting

Whole-cell lysates were prepared using RIPA buffer [50 mM Tris (pH 7.4), 2 mM EDTA, 150 mM NaCl, 0.1% SDS, and 1.0% Triton X-100] supplemented with Halt phosphatase and protease inhibitor cocktails (Fisher Scientific, Waltham, MA). The protein was quantified using the Pierce BCA Protein Assay (Fisher Scientific) as per the manufacturer’s instructions. The protein (25 µg) was subjected to SDS-PAGE, transferred to PVDF membranes, and blocked with 5% non-fat dried milk in 1X TBS-T (Tris-buffered saline with 0.1% Tween-20) for 1 h at room temperature (RT), and then a primary antibody recognizing DNA-PKcs (Abcam) was added overnight at 4°C in blocking buffer. Membranes were washed, and then an HRP-conjugated secondary antibody (Jackson ImmunoResearch, West Grove, Pennsylvania) was added for 1 h at RT. The DNA-PKcs protein was assessed using the Clarity Western ECL Substrate (Bio-Rad, Hercules, CA) and imaged using a ChemiDoc MP Imaging System (Bio-Rad). Total vinculin levels (Santa Cruz) were assessed as a loading control. To validate the expression of selected proteins, cells were treated with DMSO (0.1% v/v) or etoposide for 24 h. Whole-cell lysates were prepared as mentioned above. To detect ATAD2 and ARRB1, the following primary antibodies were used at a 1:1000 dilution: anti-ATAD2 (Cell Signaling) and anti-ARRB1 (Cell Signaling) in recommended blocking buffer. The concentration of the secondary antibody was applied as described above.

### Clonogenic survival assay

We assessed clonogenic survival as previously described (Joshi et al., 2019). Plating efficiency (PE) and surviving fraction (SF) were calculated: PE = (number of colonies formed divided by the number of cells seeded) ×100; SF = PE of treated cells divided by PE of control cells. Each data point is the average of three independent biological replicates.

### Cell viability

Cells were cultured in complete media in white-walled 96-well plates (CoStar, Corning, NY) for 24 h, treated with increasing concentrations of etoposide or DMSO, and then incubated for 72 h. Cell viability was quantified using the CellTiter-Glo Assay (Promega, Madison, WI) as per the manufacturer’s instructions.

### RNA isolation

Cells were treated with DMSO (0.1% v/v) or 20 µM etoposide for 24 h. Total RNA from three biological replicates of genotypes or treatments were isolated using the TRIzol reagent (Millipore Sigma). RNA was dissolved in nuclease-free water followed by spectrophotometric analysis of the RNA quantity; RNA was subjected to electrophoresis on an agarose gel to assess RNA quality and purity.

### Transcriptome library preparation and sequencing

RNA libraries were constructed using the NEBNext Ultra RNA Library Prep Kit for Illumina following the manufacturer’s instructions (NEB, Ipswich, MA, USA). Briefly, mRNAs were first enriched with Oligo (dT) beads. Enriched mRNAs were fragmented for 15 min at 94°C. First-strand and second-strand cDNAs were subsequently synthesized. cDNA fragments were end-repaired and adenylated at 3′ ends, and universal adapters were ligated to cDNA fragments, followed by index addition and library enrichment by limited-cycle PCR. The sequencing libraries were validated on the Agilent TapeStation (Agilent Technologies, Palo Alto, CA, USA) and quantified by using the Qubit 2.0 Fluorometer (Invitrogen, Carlsbad, CA) as well as by quantitative PCR (KAPA Biosystems, Wilmington, MA, USA). The sequencing libraries were then clustered on a single lane of a flow cell. After clustering, the flow cell was loaded on the Illumina HiSeq instrument (4000 or equivalent) according to the manufacturer’s instructions. The samples were sequenced using a 2 × 150 bp paired-end (PE) configuration. Image analysis and base calling were conducted by HiSeq Control Software (HCS). Raw sequence data (.bcl files) generated from Illumina HiSeq were converted into fastq files and de-multiplexed using Illumina’s bcl2fastq 2.17 software. One mismatch was allowed for index sequence identification.

### Differentially expressed gene analysis

The low-quality raw reads (fastq format) were filtered based on the Q30 and GC content, and then the Illumina adapters were trimmed of reads for a minimum read length of 36 bases using Trimmomatic v0.34 (Bolger et al., 2014). The index of the Chinese hamster reference genome (CHOK1GS_HDv1) was built using HISAT2 v2.1.0 (Kim et al., 2015). The Hisat2 v2.2.1 tool was used to align the reads to the genome sequences in the FASTA format, and the output aligned reads in the binary alignment map (BAM) format were translated into the transcriptomes of each sample using the StringTie v2.0 tool, which uses a novel network flow algorithm as well as an optional *de novo* assembly step to assemble and quantitate full-length transcripts representing multiple splice variants for each gene locus (Pertea et al., 2015). The StringTie outputs (GTF files) were merged to create a single master transcriptome GTF with the exact same naming and numbering scheme across all transcripts. The feature count tool implemented under Subread v2.0 was used to quantify transcripts assembled by StringTie mapped to each gene (Liao et al., 2014). Eventually, the differentially expressed (DE) gene profiles were statistically analyzed through edgeR and limma R libraries (Robinson et al., 2010; Ritchie et al., 2015).

### Biological pathway analysis

The ClusterProfiler R tool was applied on the DEGs to demonstrate the functional pathways and gene network enrichment analysis (Yu et al., 2012). This analysis provides the information related to the biological pathways significantly enriched in up or downregulated genes through mediating the Kyoto Encyclopedia of Genes and Genomes (KEGG) database and Gene Ontology (GO) terms (using a standard false discovery rate (FDR) <0.05 and *p*-value cutoff 0.05).

### Regulatory network analysis

The upstream regulator network analysis was performed through the ingenuity pathway analysis (IPA, QIAGEN) platform. The IPA casual network approach was applied on contrast matrices, by having gene symbol, log fold change, and *p*-value <0.05 of each gene, to characterize the upstream regulators as well as master regulators (the root of network) responsible for driving a set of target genes (Krämer et al., 2014).

### qPCR

RNA-Seq data were validated by qPCR from a new set of RNAs isolated from the CHO cells treated with the same treatment or control. We selected 14 master regulators based on their function in inflammatory regulation, neuronal development, and chromatin modification, including CXCR3, HIF1, IGFBP2, IL17RA, FLT1, IL1B, IL20, SPP1, STAT5, KDR, PDGF-BB, TNF, VEGF, and VEGFA. We selected 17 differentially expressed genes regulated by those master regulators and are involved in the functions we were interrogating. Primers were designed from the selected mRNA sequences using the Pickprimers tool of NCBI. Sequences of all primers are listed in Table 1. All primers were validated for qPCR using iTaq Universal SYBR Green Supermix (Bio-Rad) and the CFX Connect Real-Time PCR Detection System (Bio-Rad). RNA from three biological replicates was isolated using the TRIzol reagent (Millipore Sigma) and DNA removed with the TURBO DNA-free™ Kit (Ambion). cDNA was synthesized from 1 μg mRNA using an iScript cDNA Synthesis Kit (Bio-Rad) according to the manufacturer’s instructions. Twenty nanograms of cDNA was subjected to qPCR according to the manufacturer’s protocol. Glyceraldehyde-3-phosphate dehydrogenase (GAPDH) was used as a housekeeping gene, and the relative expression was analyzed by Cq values of the target and control genes using the 2-delta delta Ct method (Livak and Schmittgen, 2001).

**TABLE 1:** Primer sequences of select genes with fold changes observed during mRNA sequencing analysis.

Gene	Primer	Primer sequence (5′ —> 3′)	LogFC in mRNA-seq data			
			KR-DvsWT-D	KR-EvsWT-E	WT-EvsWT-D	KR-EvsKR-D
ARRB1	ARRB1-Fwd	GAT​CTT​GCA​TCC​AGT​GAT​GTG​G	2.17	1.88	1.43	1.34
	ARRB1-Rev	GAT​GTG​GGG​GCT​CCT​CTT​TC				
ASPM	ASPM-Fwd	TGT​GAG​CCA​CAT​CCA​GAC​AC	2.5	2.32	0.55	0.37
	ASPM-Rev	AGT​TCC​ATG​GTT​CGC​ACG​AG				
ATAD2	ATAD2-Fwd	GTC​GAG​TCA​CAT​TGC​AGC​AC	2.17	1.61	1.35	0.79
	ATAD2-Rev	AAA​ACA​AAC​AAC​CTC​TGG​GGG				
CCDC88A	CCDC88A-Fwd	GCA​TCA​CTG​CAG​CAT​CTA​ATG​T	2.5	2.78	-	0.37
	CCDC88A-Rev	AGG​AGC​TTG​GAT​GCT​CCC​TA				
CENPF	CENPF-Fwd	TTT​ACA​ACT​CCA​CTC​ACA​CCA​A	3.95	3.86	0.58	0.49
	CENPF-Rev	GGC​TGG​CTC​ACG​TTT​TTA​GC				
CNTF	CNTF-Fwd	GCA​ACT​GGT​CGG​TCT​TGG​TT	-	2.29	-	-
	CNTF-Rev	TCC​CCT​AAA​TCA​ACC​TGG​GG				
DCN	DCN-Fwd	CTC​CTT​TCC​ACA​CCT​GCA​AAC	−3.06	−2.53	−0.19	0.32
	DCN-Rev	TAC​TTT​TAC​AAC​CTG​GGA​ACC​TTT​T				
GAP43	GAP43-Fwd	GCT​GAG​GAA​GAG​AAA​GAA​GCT​GTA	0.14	2.27	0.43	2.56
	GAP43-Rev	CCT​CGG​GGT​CTT​CTT​TAC​CC				
IQGAP2	IQGAP2-Fwd	GAG​AGA​CGC​GTA​TGA​GGA​GC	2.06	1.24	0.8	-
	IQGAP2-Rev	CAC​AGC​AGC​CAG​CCT​ATT​GA				
KIF18A	KIF18A-Fwd	GGG​AAG​ACT​CAC​ACG​ATG​CT	2.55	2.07	0.86	0.39
	KIF18A-Rev	CCT​CTG​AGG​ATT​TAG​GCT​CCA​A				
KIF20B	KIF20B-Fwd	AGT​TGG​AAC​TGA​AGA​AGC​GTG	3.16	3.01	0.75	0.61
	KIF20B-Rev	TGA​GCA​AGT​TCA​GCC​TGT​TTC				
MKI67	MKI67-Fwd	CAC​CTT​GCT​CCA​GAT​AAG​AGT	2.09	1.63	0.63	-
	MKI67-Rev	AAT​TCA​GGT​CTT​AGA​CGA​CCA​CC				
SMC2	SMC2-Fwd	TTG​ACC​CCC​TCT​TCA​ATG​CT	2.6	2.33	0.69	0.42
	SMC2-Rev	ATT​AGA​AGC​CCG​CAC​CTG​AG				
SMC4	SMC4-Fwd	CTG​GAG​CTC​CTC​GTC​TAA​TG	2.74	2.86	-	0.34
	SMC4-Rev	ATA​ATA​CAG​GAA​AAG​CGC​TTA​TGG​A				
UNC5B	UNC5B-Fwd	CAT​CCG​CAT​TGC​TTA​CCT​GC	−1.86	−2.2	−0.59	−0.94
	UNC5B-Rev	GCA​CTG​CAG​AAG​GAC​CTC​AT				
VCAM1	VCAM1-Fwd	CCT​TCA​TTC​CTA​CCA​CCG​AAG​A	2.22	2.87	0.35	1
	VCAM1-Rev	ATT​TCC​CTG​GGG​GCA​TCG​TT				

WT-D, WT cells treated with DMSO; WT-E, WT cells treated with etoposide; KR-D, KR cells treated with DMSO; KR-E, KR cells treated with etoposide.

### Statistical analysis

Cell viability was analyzed using non-linear regression function in GraphPad Prism (version 8; San Diego, CA) comparing viability as a function of the log etoposide dose. Data points represent an average of the three biological replicates. The area under the curve (AUC) for viability and colony-forming assays was assessed using GraphPad Prism, and the resultant total peak AUC and standard error per sample were compared using one-way ANOVA with an *ad hoc* Tukey’s multiple comparisons test. qPCR results were assessed using two-way ANOVA with Fisher’s LSD test to detect the significant differences among the mean relative expression levels.

## Results

### DNA-PKcs promotes survival following exposure to etoposide

**FIGURE 1:**
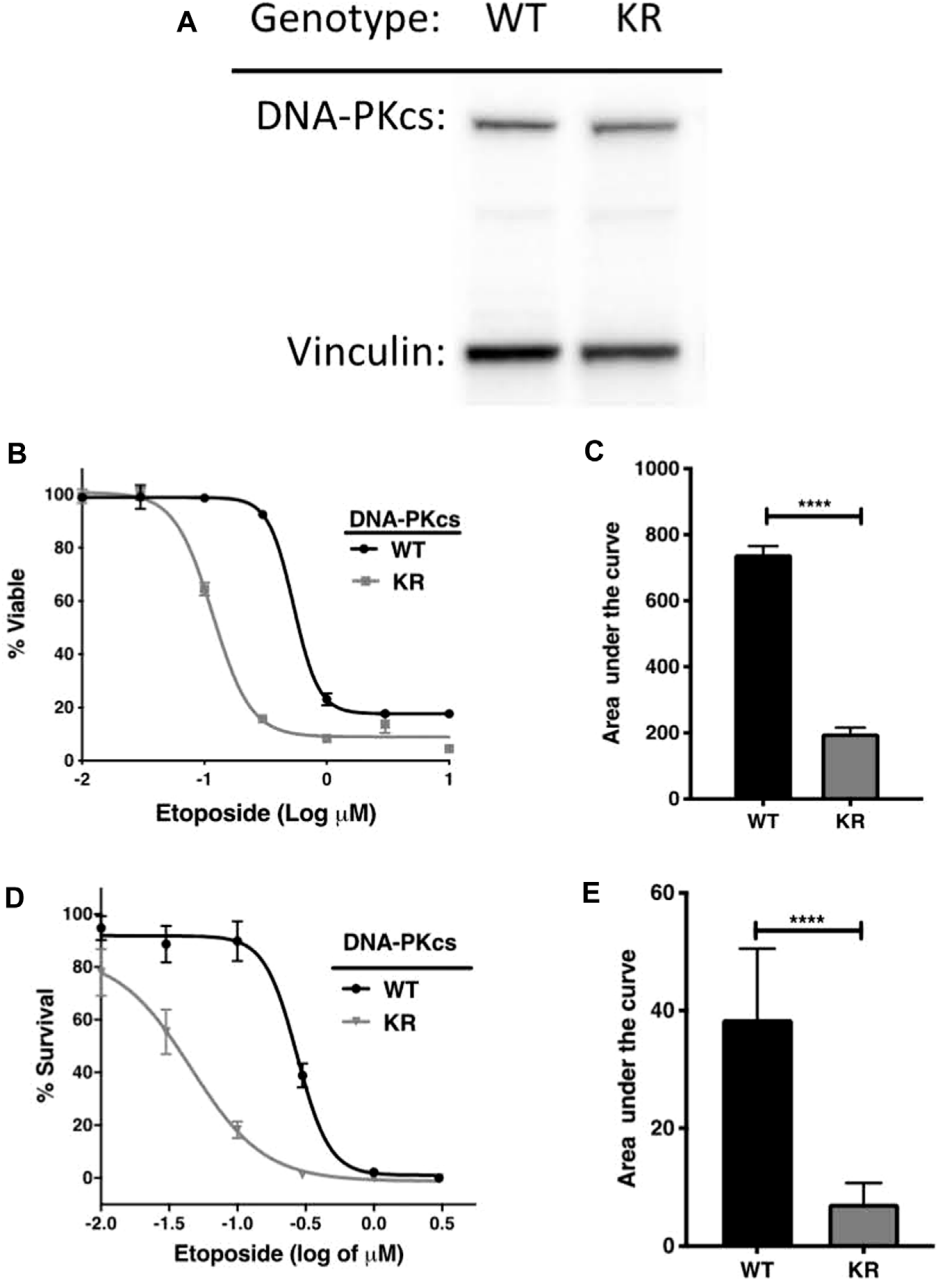
DNA-PKcs protects cells from etoposide toxicity via its kinase domain. **(A)** Evaluation of DNA-PKcs proteins in CHO variants by Western blot. **(B, C)** Cell survival profiles of CHO cells assessed using the CellTiter-Glo (CTG) assay with increasing doses (0.003–1 μM) of etoposide at 72 h post-exposure with the associated area under the curve analysis. **(D, E)** Clonogenic survival of DNA-PKcs WT or KR cells following exposure to etoposide (0.003–1 μM) with the associated area under the curve analysis, ****p < 0.0001.

We confirmed DNA-PKcs production by Western blotting on WT and KR CHO cells. DNA-PKcs is produced equally in both WT and KR cells (Figure 1A). Then, we assessed the effect of DNA-PKcs on cell survivability after exposure to the topoisomerase II inhibitor, etoposide. Consistent with previous reports, cells lacking DNA-PKcs kinase activity (KR cells) are more sensitive to etoposide (Figures 1B–E) (Convery et al., 2005).

### Differential expression mediated by DNA-PKcs

**FIGURE 2:**
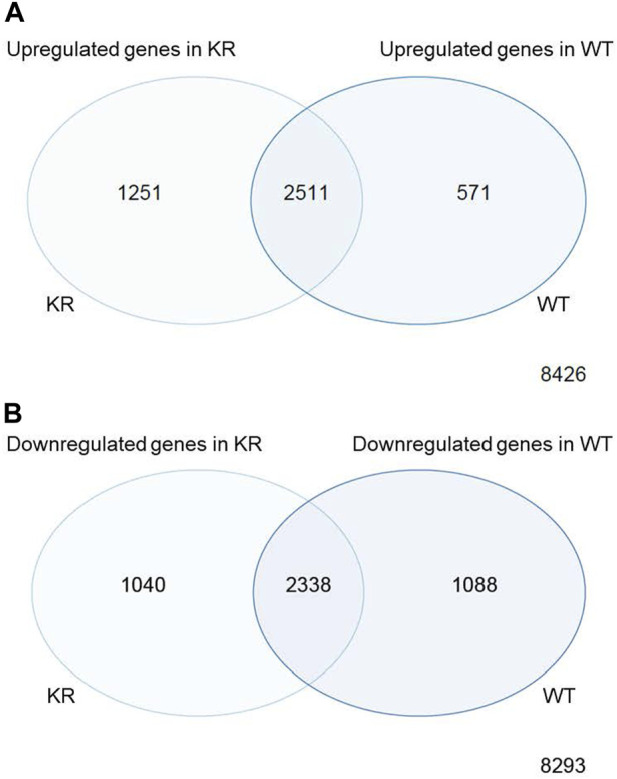
Comparison of shared versus discreet differentially regulated genes in DNA-PK wild-type (WT) versus kinase-inactive (KR) cells. **(A)** Shared or exclusively upregulated genes in KR and WT cells after etoposide exposure. **(B)** Shared or exclusively downregulated genes in KR and WT cells after etoposide exposure.

We assessed the replicates of two isogenic CHO genotypes, WT and KR, treated with either DMSO or etoposide by principal component analysis (PCA; Supplementary Figure S1). All replicates within the genotype and treatment combinations (such as WT–DMSO, WT–etoposide, KR–DMSO, or KR–etoposide) clustered closely, indicating that the replicates strongly correlated with one another. A total of 12,759 genes were of sufficient quality to allow assessment of potential differential regulation in our samples (Table 2). In each of our comparisons, approximately 7,000 genes were differentially expressed, either up or downregulated. We assessed genotype-specific differentially expressed genes (DEGs) by comparing DMSO-treated WT versus KR gene expression. Etoposide-induced alterations within each genotype were compared to those induced by DMSO alone, and finally, differential expression due to drug treatment in WT was compared to that in KR cells. Each red dot represents a single gene with a significant (*p* < 0.05) positive log-fold change, and the blue dot denotes a gene with a significant negative log-fold change (Supplementary Figures S2A–D). Upon assessing DEGs in KR following etoposide exposure to those in WT, we found most genes are induced independent of DNA-PKcs kinase activity, as these were upregulated in both KR and WT (Figure 2A), where 571 of the upregulated genes were discretely induced in WT and 1,251 were observed only in KR cells. In comparing the diminished expression, 2,338 of 4,466 genes were downregulated independently of DNA-PKcs kinase in both KR and WT in response to etoposide exposure (Figure 2B). The number of privately downregulated DEGs is similar in WT and KR cells (Figure 2B).

**TABLE 2:** Number of genes identified for assessment of potential differential regulation based on genotype.

Comparison	Numbers of genes downregulated	Numbers of genes upregulated	Total Numbers of DEG	Numbers of genes with no change	Total Numbers of genes
Genotype-specific alterations (DMSO only), WT-D vs. KR-D	3,818	3,798	7,616	5,143	12,759
Genotype-specific alterations (etoposide only), WT-E vs. KR-E	3,285	3,604	6,889	5,870	12,759
Etoposide-induced alterations by genotype					
WT-E vs. WT-D	3,426	3,082	6,508	6,251	12,759
KR-E vs. KR-D	3,378	3,762	7,140	5,619	12,759

### DNA-PKcs alters the transcription of multiple genes involved in various cellular processes

**FIGURE 3:**
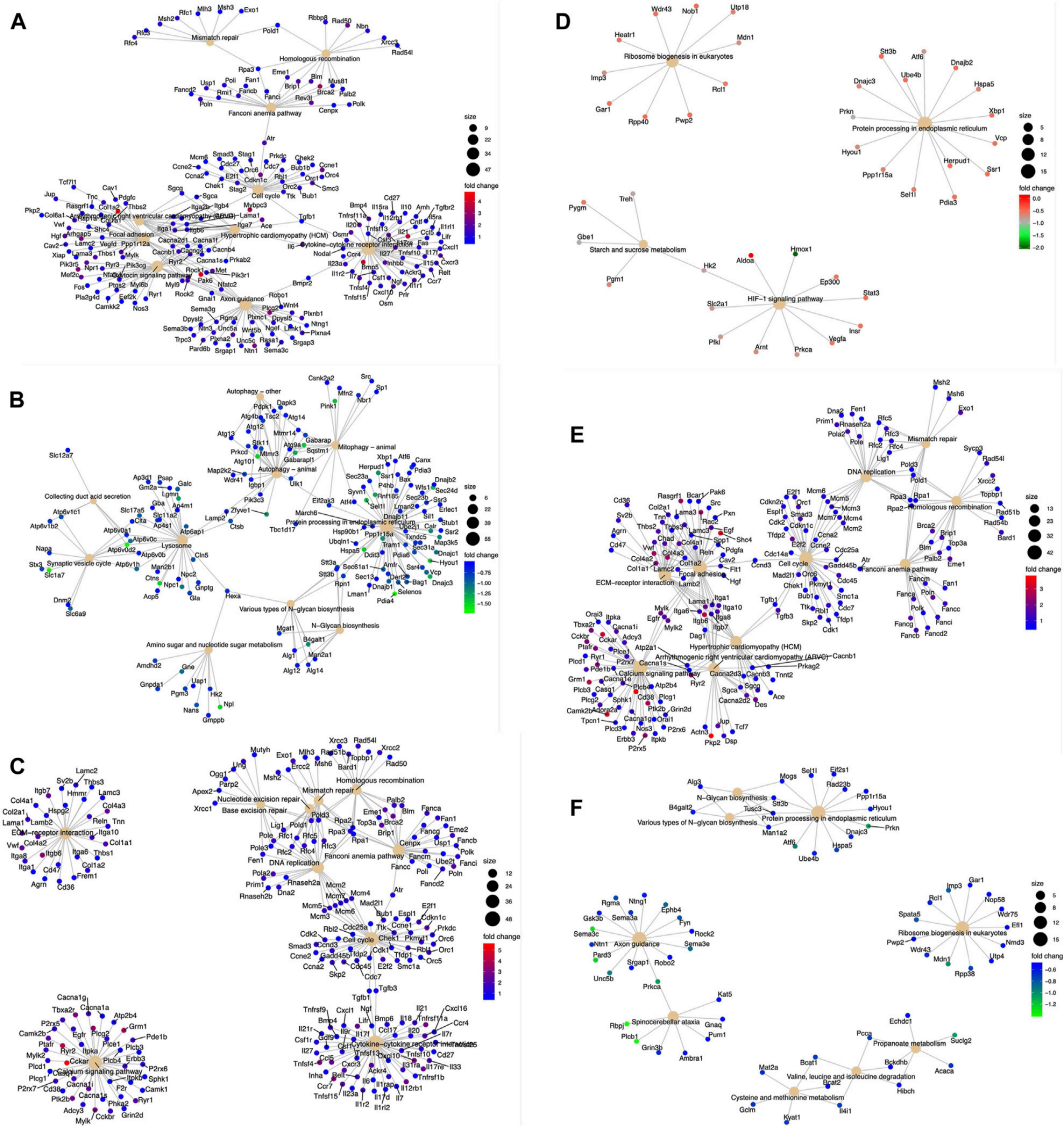
Diverse biological pathways regulated by the DEGs from the mRNA seq data found from KEGG pathway analysis. Upregulated genes are represented with red (highest positive fold change) and blue (lowest positive fold change) dots, and downregulated genes are represented with blue (lowest negative fold change) and green (highest negative fold change) dots. Corresponding pathways regulated by DEGs are represented with a solid tangerine circle. The size of the correlates with the number of genes. **(A**, **B)** Upregulated and downregulated, respectively, genes in KR cells compared to WT; **(C, D)** upregulated and downregulated, respectively, genes in WT cells after etoposide exposure; **(E, F)** upregulated and downregulated, respectively, genes in KR cells after etoposide exposure.

DEGs were analyzed with the KEGG pathway database and were involved in diverse cellular processes (Figures 3A–F). We analyzed these pathways regulated by DEGs to ascertain significantly altered biological processes within WT and KR cells in control (DMSO) or DNA-damaging conditions. We compared the biological pathways populated by the DEGs in WT and KR cells without etoposide treatment. A total of 14 biological pathways were enriched in upregulated DEGs (Supplementary Figure S3A), and seven pathways were associated with downregulation (Supplementary Figure S3B) in KR cells compared to WT. We observed that several genes involved in DNA damage repair pathways were upregulated in KR cells (Figure 3A and Supplementary Figure S3A). Genes upregulated in KR cells compared to WT cells were predominantly involved in cell cycle, cytokine signaling and associated anomalies, and cytoskeletal regulation (Supplementary Figure S3B). Genes upregulated in KR compared to WT were involved with arrhythmogenic right ventricular cardiomyopathy (ARVC), hypertrophic cardiomyopathy (HCM), focal adhesion, axon guidance, and cytokine signaling pathways, indicating DNA-PKcs kinase-dependent regulation of these pathways. In contrast, genes involved in protein processing and degradation, autophagy, and protein and sugar metabolism were less abundant in KR cells than in WT cells (Figure 5B; Supplementary Figure S3B).

### Etoposide-induced differentially regulated biological pathways within WT and KR cells

**FIGURE 4:**
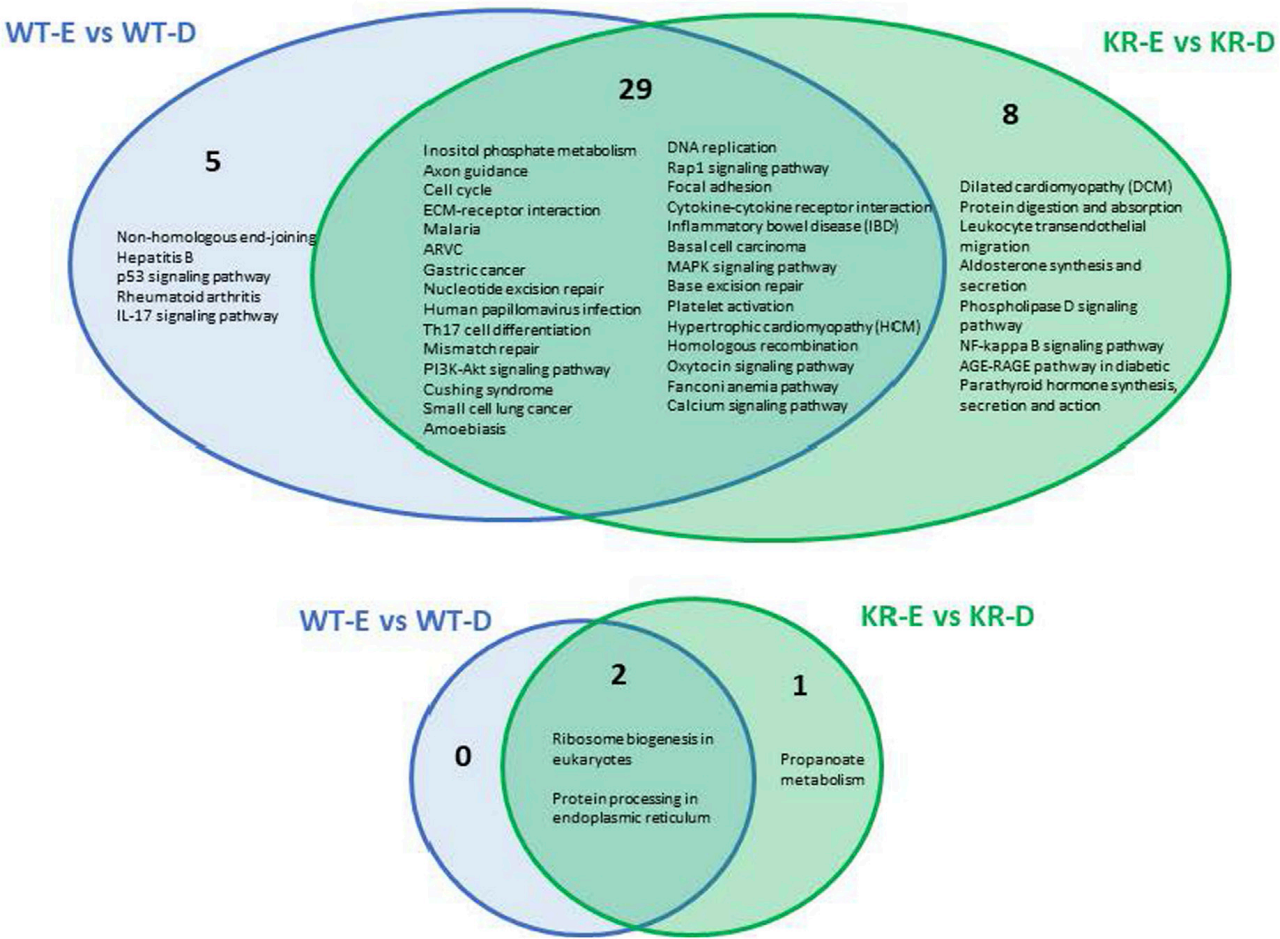
Comparison of shared versus divergent etoposide-induced biological pathways altered by differential gene regulation in DNA-PKcs wild-type (WT) compared to kinase-inactive (KR) cells. Etoposide-induced upregulated biological pathways in WT and KR cells. Etoposide-induced downregulated biological pathways in WT and KR cells.

We analyzed the role of DNA damage in differentially regulated biological pathways between cell lines. A total of 34 pathways were upregulated following etoposide treatment in WT cells (Supplementary Figure S4A), whereas only two pathways were suppressed in WT cells (Supplementary Figure S4B). In KR cells, 37 pathways were upregulated (Supplementary Figure S4C) and three pathways were downregulated (Supplementary Figure S4D) after etoposide treatment. Comparing KR to WT following drug treatment, five pathways were uniquely upregulated in WT, and eight were observed only in KR following drug treatment. Out of 37 pathways, 29 were shared between genotypes, (Figure 4; Supplementary Figures S4A and C) indicating that genes represented here are upregulated independent of DNA-PKcs kinase activity. Three pathways decreased in response to etoposide treatment, in which two were observed in both cell lines, again indicating they are regulated in a DNA-PKcs kinase-independent manner (Figure 4; Supplementary Figures S4B and D). Multiple DNA damage repair processes were commonly upregulated in both cell types after etoposide treatment, whereas NHEJ was exclusively induced in WT cells (Figure 4). Other commonly upregulated pathways including cell cycle, DNA replication, cytokine signaling, calcium signaling, and extracellular matrix (ECM)–receptor interactions are observed in both WT and KR cells. The two common pathways that were downregulated in both WT and KR cells were related to ribosome biogenesis and protein processing (Figure 4).

### Differentially regulated biological processes between etoposide-exposed WT and KR cells

**FIGURE 5:**
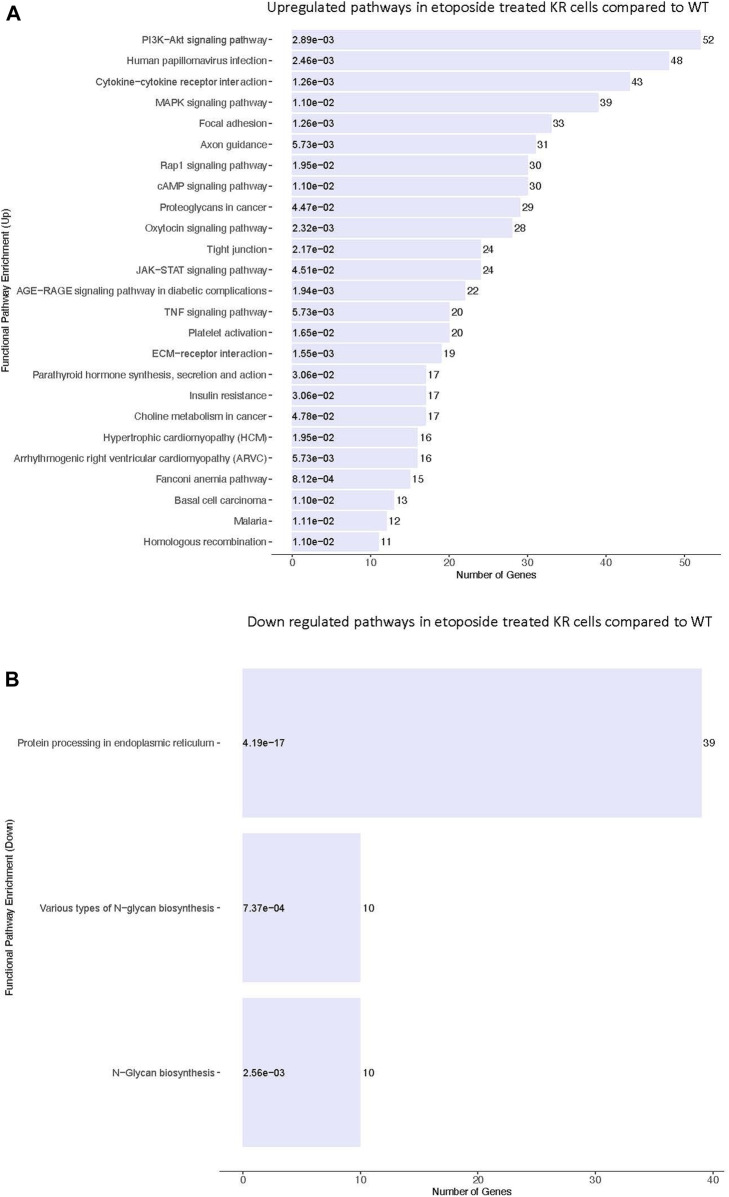
Comparative assessment of etoposide-induced biological pathways altered by differential gene regulation in DNA-PKcs wild-type (WT) and kinase-inactive (KR) cells. **(A)** Upregulated biological pathways in etoposide-treated KR cells compared to WT genotype-specific upregulated biological processes. **(B)** Downregulated biological pathways in etoposide-treated KR cells compared to WT.

**FIGURE 6:**
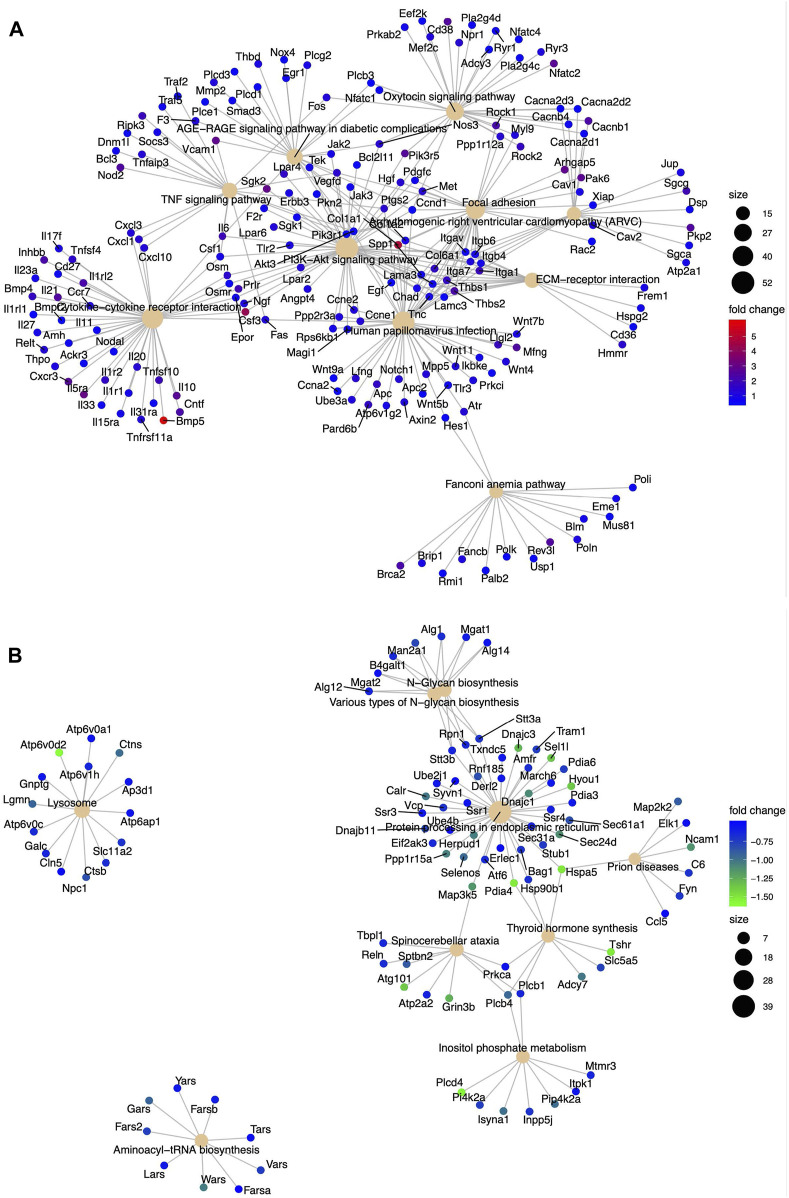
Diverse biological pathways regulated by the DEGs from the mRNA seq data found from KEGG pathway analysis in etoposide-treated WT and KR cells. Upregulated and downregulated genes are represented with blue and red dots and blue and green dots, respectively. Pathways are shown as a solid tangerine circle. **(A, B)** Upregulated and downregulated, respectively, genes in etoposide-treated KR cells compared to WT.

We assessed the biological pathways impacted by differential gene regulation between each genotype after exposure to etoposide to discern how DNA-PKcs regulates expression changes following topoisomerase II inhibition (Figures 5A, B). When comparing the effects of etoposide on gene expression between genotypes, we found several genes upregulated in KR cells compared to WT cells in a kinase-dependent and etoposide-independent fashion. These upregulated genes were involved in multiple kinase-driven signaling pathways, including many involved in cancer biology, such as TNF signaling, cell–cell and cell–matrix interactions, and DNA damage response pathways. Genes involved in pathways related to human papilloma virus infection were also upregulated in etoposide-treated KR cells compared to etoposide-treated WT cells (Figure 6A). Downregulated genes in KR cells compared to WT after etoposide exposure are involved in mainly protein processing and carbon metabolism (Figure 6B).

### Analysis of global transcriptional regulators

We evaluated our DEGs using ingenuity pathway analysis (IPA, ^©^ 2000–2020, QIAGEN) to determine which master regulators are driving gene expression changes observed in our samples. Master regulators possibly activated or inactivated in response to etoposide exposure to induce or suppress the DEGs are listed in Table 3. A complete summary of IPA-designated master regulators is presented in Supplementary Tables S1–S4. Master regulators controlling genes induced in KR cells without treatment include the estrogen receptor, growth hormone, IGFBP2, and corticotropin-releasing hormone receptor. We also noted an enrichment in proteins involved in proliferation and genes associated with the inflammatory response and angiogenesis. Master regulators diminishing expression include potential tumor suppressors and known modulators of gross or intracellular morphology. IPA reveals that genes regulated by drugs inhibiting VEGF and various pro-growth pathways, including those induced by oncogenes such as Raf, KIT, ALK, and ROS1, are diminished. Overall, this indicates that the cellular milieu induced by expressing DNA-PKcs without functional kinase activity varies substantially compared to that of WT. We analyzed etoposide-induced DEGs in both cell types for detecting the master transcriptional regulators that modify DNA damage-related gene expression changes in WT or KR cells after etoposide exposure (Table 2) Ten master regulators were detected commonly in etoposide-treated cells, whereas most were discreet to each cell type. We contrasted the master regulators between etoposide-treated WT and KR cells. Comparing the upregulated genes in etoposide-treated KR cells to WT cells, we found 14 global regulators, and four upstream global regulators dictated gene repression in the KR.

**TABLE 3:** Summary of shared or discrete master regulators identified in cells expressing wild-type (WT) or kinase-inactive (KR) DNA-PKcs with or without etoposide exposure.

Activated in response to etoposide			Inhibited in response to etoposide		
KR	WT	SHARED	KR	WT	SHARED
CALR	CCNE1	CBX4	AHRR	FOXH1	CDKN2A
CAND1	E2F1	CCND1	CBFA2T3	FOXP3	DNMT3L
CREB1	E2F3	CTNNB1	CDKN2C	GLIS2	E2F6
EBF2	ETV1	E2F2	ETV6	HDAC1	ETS1
EHMT1	FOXM1	EHF	HOXA4	HDAC7	HDAC11
ELK1	HDAC2	KDM3A	IKZF2	HEYL	RBL1
ETV7	HOXA9	MITF	MAFK	HOXA4	SOX11
FOXF1	NOTCH3	NFIC	NEUROG1	MEOX1	SPDEF
FOXL2	SERTAD1	PURA	NEUROG2	NFX1	ZIC2
GATA2	SMARCE1	REL	NONO	NOSTRIN	
HES1	SP3	SMAD2	TOB1	NUPR1	
LIMD1	TAL1	SMARCA4	TP73	PSIP1	
MEF2C	TCF7L2	TBX2	TRIM24	PTTG1	
MRTFB	TP63	TFDP2	ZFP36	TRERF1	
MYB	UHRF1	TP63	ZGPAT	ZFHX3	
NFAT5	WBP2	YAP1		ZFP36	
NOTCH4	ZNF217				
SCML2	ZNF281				
SMAD5					
SNAI1					
SOX2					
SOX7					
TAF4					
TAF4B					
THAP12					

### Relative expression analysis of selected genes by qPCR and immunoblotting

**FIGURE 7:**
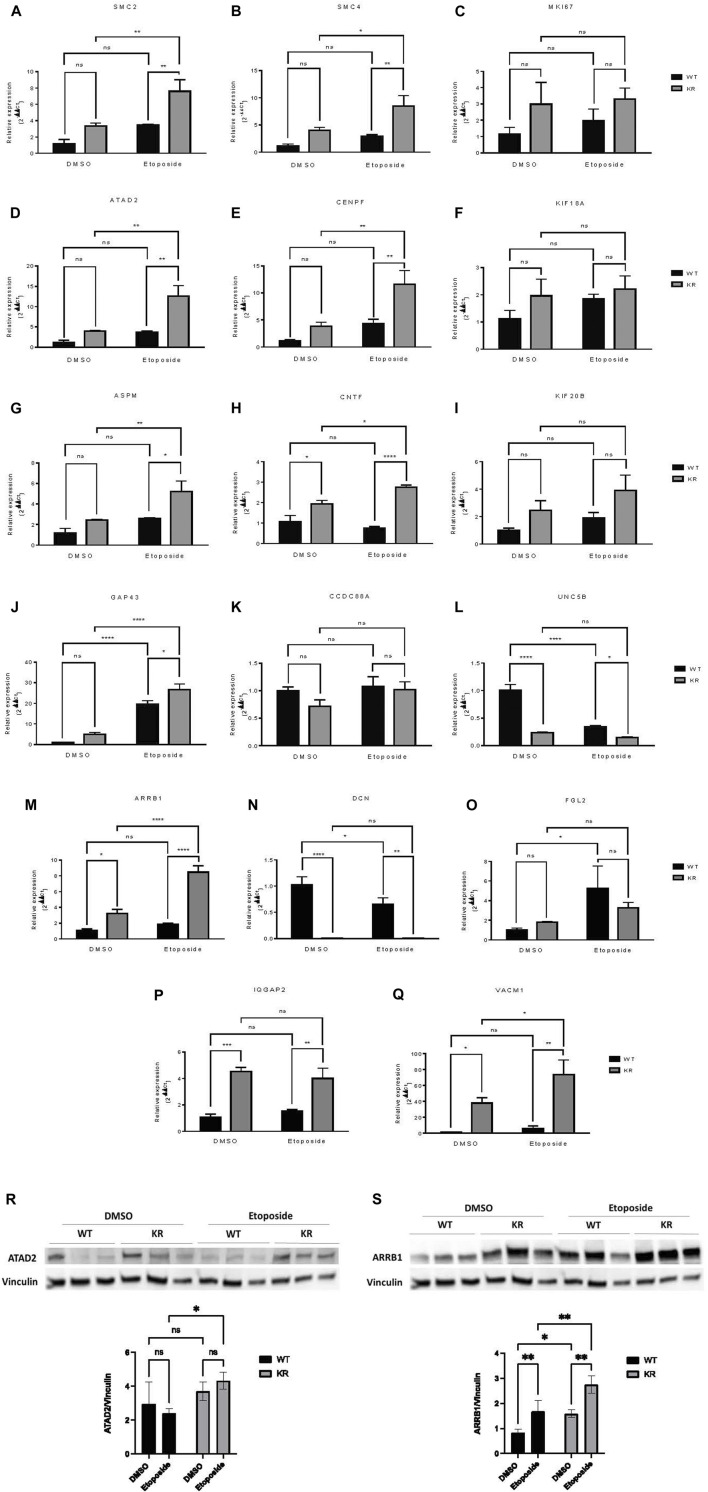
Relative expressions of selected genes detected by qPCR and immunoblotting showed a similar trend with mRNA sequencing data. **(A–Q)** Seventeen DEGs were selected to perform qPCR to detect their relative expression in WT and KR cells with or without etoposide treatment. The mean relative expression of a DEG was analyzed by Cq values of the target and control genes using the 2^−ΔΔCq^ method after normalizing with the Cq value of GAPDH for any specific sample. **(R, S)** Two DEGs were selected for immunoblotting to detect their protein expression in WT and KR cells with or without etoposide treatment. Two-way ANOVA with Fisher’s LSD test was used to detect the significant differences among the mean relative expression levels. ns: non-significant, *p < 0.05, **p < 0.01, ***p < 0.001, and ****p < 0.0001.

Seventeen DEGs were selected for qPCR based upon the above data. The mean relative expression of DEGs was calculated from the Cq values from the qPCR (Figures 7A–Q). For all genes, the relative expression pattern detected by qPCR followed the same trend observed in RNA-Seq (Table 3). Furthermore, two genes were selected for immunoblotting to validate the protein expression in WT and KR cells (Figures 7R, S). Overall, the trends supported our findings in RNA-Seq and indicated that the kinase activity of DNA-PKcs differentially affects gene regulation with and without exogenous DNA damage.

## Discussion and conclusion

DNA-PKcs is a key regulator in two major DNA DSB repair pathways, HR and NHEJ, and is an emerging therapeutic target for multiple cancer subtypes, with most inhibitors preventing its kinase activity (Blackford and Jackson, 2017; Mohiuddin and Kang, 2019; Dylgjeri and Knudsen, 2022). DNA-PKcs is also involved in the phosphorylation of many transcriptional regulators (Collis et al., 2005; Goodwin et al., 2015; Park et al., 2017). We used RNA-Seq to analyze mRNA transcripts isolated from DNA-PKcs WT and kinase-inactivated KR CHO cells after exposure to etoposide. We analyzed our RNA sequencing data and identified the differentially regulated biological pathways that depend on DNA-PKcs kinase activity with or without DNA damage. Previous studies indicated a limited role of DNA-PKcs in transcription after DNA damage; however, data from these studies are not directly comparable to RNA-Seq data (Galloway and Allalunis-Turner, 2000; Bryntesson et al., 2001; An et al., 2005; Goodwin et al., 2015). Novel findings from the current study revealed more than 7,000 DEGs in KR cells compared to WT, while etoposide treatment alone changed the expression of more than 6,500 and 7,100 genes in WT and KR, respectively.

Upregulated genes in DMSO-treated KR cells compared to WT were enriched in three DNA damage repair pathways, including HR. DNA-PKcs is a core component of NHEJ; thus, cells lacking catalytically active DNA-PKcs rely more heavily on HR to repair endogenously formed DSB. We also observed the upregulation of several genes involved in cell cycle regulation and proliferation, in agreement with our previous work, indicating that DNA-PKcs regulates replication in a kinase-dependent fashion in replication stress (Liu et al., 2012; Ashley et al., 2014). Genes upregulated in KR cells compared to WT cells are involved in cell cycle, ARVC, and HCM, indicating that DNA-PKcs regulates gene expression without DNA damage or that these are upregulated in a compensatory manner. Downregulated genes in KR compared to WT cells regulate protein and sugar metabolism (Figure 3B). Goodwin et al. (2015) previously reported that overexpression of various metabolic pathways in DNA-PKcs depleted cancer cells. However, our results found downregulation of genes associated with metabolic pathways in the absence of DNA-PKcs kinase.

We observed that 29 biological pathways were commonly upregulated in both WT and KR cells (Supplementary Figure S4) in a DNA-PKcs kinase-independent manner. Several genes involved in the DNA damage response and inflammatory signaling were upregulated in etoposide-treated KR cells alone, indicating their transcription may be regulated by the kinase activity of DNA-PKcs. Differences in HR-based repair are noted in the WT versus KR cells following exposure to other genotoxic agents, as KR cells promote higher levels of recombination compared to WT cells or cells lacking DNA-PKcs (Ashley et al., 2014).

Etoposide exposure modified the global transcription of several genes in both genotypes. Genes involved in NHEJ are discreetly upregulated in WT cells in the presence of fully functional DNA-PKcs. Genes associated with various DNA repair pathways along with cell cycle and DNA replication were upregulated in both cell lines. Ribosome biogenesis transcripts were downregulated independently of DNA-PKcs, consistent with previous reports which suggest that several DNA repair proteins have a defined role in ribosome biogenesis (Ogawa and Baserga, 2017). Upregulated genes in KR may help the DNA-PKcs kinase-deficient genotypes to survive in the absence of a functional classical NHEJ. Genes involved in inflammation were repressed in WT after etoposide exposure but induced in KR cells. Chronic proinflammatory factors are correlated with persistent DNA damage and induce the expression of IFN-regulated genes (Brzostek-Racine et al., 2011; Karakasilioti et al., 2013). DNA-PKcs kinase may alter the inflammatory milieu via impacting the stability of Egr1, which modulates proinflammatory signaling in T-cells (Waldrip et al., 2021). The robust inflammatory response to DNA damage observed in KR indicates that treatment with a DNA-PKcs inhibitor in combination with chemotherapy or radiotherapy may promote secondary tumors. Further investigations into the clinical ramifications of these data will clarify if DNA-PKcs mitigates the inflammatory response following DNA damage.

In KR, the changes in gene expression were dominantly regulated by the Akt or Wnt/β-catenin signaling pathways, as well as chromatin or developmental modulators (Table 2). Changes in genes dependent on Wnt signaling was more frequently observed in WT cells, indicating DNA-PKcs may regulate this pathway, which is consistent with our previous findings (Gurley et al., 2017). The abundance of regulators associated with development, differentiation, and steroid signaling is likely due to the origin of the cells from ovaries. However, we have previously indicated a role for DNA-PKcs in differentiation and stemness, so these observations warrant additional investigation (Gurley et al., 2017). Master regulators involved in histone modification were found in response to etoposide-mediated DNA damage. DNA-PKcs phosphorylates histone variant H2AX and promotes chromatin decondensation immediately after DSB (Lu et al., 2019). DNA-PKcs-mediated phosphorylation of the histone core after ionizing radiation weakens the DNA-histone binding, which then facilitates the H2A.Z-H2B dimer’s incorporation into reconstituted nucleosomes (Wang et al., 2019). The pattern of changes in histone modifications indicates DNA-PKcs has a role in regulating chromatin after DNA damage.

A subset of genes associated with chromatin modification, neuronal development, and proinflammatory responses was assessed via qPCR. SMC2 and SMC4, components of condensin complex I and II, and CENPF, a component of centromere–kinetochore proteins, all required for mitotic chromosome segregation, were upregulated robustly in KR cells following damage (Figures 7A, B, E) (Varis, Salmela, and Kallio, 2006; Wu and Yu, 2012). Condensin I and II, respectively, participate in DNA single-strand damage and regulate HR-mediated DSB repair (Heale et al., 2006; Wood et al., 2008), whereas overexpression of CENPF is associated with metastasis and co-immunoprecipitates with DNA-PKcs (Du et al., 2010; Yu et al., 2020). KR cells are dependent on HR due to an inability to complete NHEJ, so induction of SMC2, SMC4, or CENPF after etoposide exposure might be expected. On the other hand, ATAD2 is required for histone hyperacetylation, facilitating progression and proliferation in multiple cancers by promoting the expression of some genes from the kinesin family (Liu et al., 2019; Zheng et al., 2015; Zou et al., 2014). Increase in ATAD2 in KR cells (Figures 7D, R) indicates its impact in surviving DNA damage in DNA-PKcs kinase inactivity, consistent with previous findings, where ATAD2 inhibition has shown promising results in inducing apoptosis and autophagy in breast cancer cells (Yao et al., 2020). All of these genes, SMC2, SMC4, CENPF, and ATAD2, could be considered potential cancer therapeutic targets for future studies.

Many genes regulating neuronal development (e.g., ASPM, CNTF, and UNC5B) were differentially regulated in a DNA-PKcs kinase-dependent manner with or without damage. ASPM regulates mitotic spindle orientation and astral microtubule dynamics in embryonic neuroblasts (Jiang et al., 2017), and significant overexpression of ASPM was observed in KR cells after etoposide exposure (Figure 7G). However, impairment of DNA DSB repair after ASPM depletion was also reported (Kato et al., 2011). Dual inhibition of DNA-PKcs and ASPM could be an interesting therapeutic strategy. CNTF is a survival factor in neuronal cells and protects cells from inflammatory destruction (Giess et al., 2002; Linker et al., 2002). CNTF was significantly upregulated in KR cells compared to WT and was further induced after etoposide treatment (Figure 7H). UNC5B is involved in NTN1-dependent cell migration and axon guidance (Shao et al., 2017; Hernaiz-Llorens et al., 2021). In breast cancer cells, UNC5B overexpression promotes proliferation and metastasis; however, it also promotes p53-dependent apoptosis (Wu et al., 2020; Arakawa, 2005). We observed a significant downregulation of UNC5B in KR cells without DNA damage (Figure 7L), and in WT cells after DNA damage; hence, DNA-PKcs may regulate UNC5B-NTN1 signaling. To the best of our knowledge, the role of DNA-PKcs in neuronal development is novel and necessitates more detailed investigation as NHEJ is the sole DSB repair pathway for post-mitotic neurons.

Finally, genes involved in proinflammatory responses were also assessed by qPCR. ARRB1 has varying impacts on NF-κB-dependent transcription (Witherow et al., 2004; Cianfrocca et al., 2014). ARRB1 and NF-κB co-expression promotes cancer progression and is correlated with poor prognosis in lung adenocarcinoma (Yu et al., 2015). High expression of ARRB1 was observed in both WT and KR cells after etoposide treatment (Figures 7M, S); thus, DNA-PKcs kinase activity dictates the etoposide-mediated response to ARRB1 gene expression. A similar pattern of expression was observed in SMC2, SMC4, ATAD2, ASPM, CENPF, and CNTF, supporting the idea that DNA-PKcs kinase activity is dictating some gene expression patterns after DNA damage through unknown mechanism(s).

DCN is a leucine-rich extracellular matrix protein which binds to TGF-β, prevents it from binding to its receptor, and may serve as an antagonistic ligand of VEGFR-2 (Khan et al., 2011). DCN induces autophagy and mitophagy in endothelial cells and breast cancer cells, respectively, and inhibits tumor cell proliferation and tumor invasiveness (Goldoni and Iozzo, 2008; Goyal et al., 2014; Neill et al., 2014). As a significant correlation has been found between the disease-free survival of patients with soft tissue sarcoma and reduced DCN expression, it is used as a prognostic marker for metastasis in breast cancer and soft tissue tumors (Matsumine et al., 2007; Goldoni and Iozzo, 2008). Low expression of DCN in KR cells (Figure 7N) indicates that DNA-PKcs’ kinase activity regulates its transcription. In normal non-tumorigenic cells, DCN promotes angiogenesis, while a high DCN level inhibits tumor vascularization (Khan et al., 2011; Mohan et al., 2011). Modified adenovirus co-expressing IL-12 and DCN have already shown potent antitumor effects in an immunogenic tumor model, indicating DCN as a promising cancer immunotherapeutic (Oh et al., 2017).

Expression of IQGAP2, a potential tumor suppressor, is reduced in human primary gastric cancer, ovarian cancer, and hepatocellular carcinoma (Jin et al., 2008; White et al., 2010; Deng et al., 2016). In prostate and ovarian cancer cell lines, IQGAP2 prevents epithelial–mesenchymal transition (EMT), thus reducing the invasiveness of cancer cells (Xie et al., 2012). IQGAP2 may have a role in the IFN-mediated antiviral immune response through NFκB (Brisac et al., 2016). *IQGAP2* ^*−/−*^ mice exhibit metabolic abnormalities (Vaitheesvaran et al., 2014). We observed a higher expression of IQGAP2 in KR cells compared to WT (Figure 7P) in an etoposide-independent fashion, indicating DNA-PKcs activity may also regulate the metabolic status and inflammation through regulation of IQGAP2 expression. This is also supported by the AGE-RAGE pathway, a central pathway in diabetes, overexpressed in KR cells compared to WT as well as in etoposide-treated KR cells (Figures 3A, 6C, 7A). VCAM1, a member of the Ig superfamily, is involved in trans-endothelial migration of leukocytes during an inflammatory response and promotes tumor cell invasiveness in breast and colon cancer (Cook-Mills, 2002; Zhang et al., 2020). Like IQGAP2, VCAM1 expression is higher in KR cells compared to the WT cells (Figure 7Q). Collectively, our data support the possibility of DNA-PKcs kinase regulation of inflammation (Figure 6A).

Overall, this global transcriptome study suggests a role for DNA-PKcs in transcriptional regulation with or without DNA damage. Our results provide additional knowledge about how DNA-PKcs modifies the transcriptional response to DNA damage, leading to alterations of many essential cellular pathways which ultimately dictate genomic stability. Multiple DNA-PKcs inhibitors are in clinical trials as potential therapeutic interventions in multiple cancer subtypes coupled with traditional chemotherapy and/or radiotherapy; however, we lack a full appreciation of how inhibition of DNA-PKcs kinase will alter the cellular transcriptional response to DNA damage. Our results indicate that impairing DNA-PKcs kinase activity may profoundly impact the transcriptional response to DNA damage, modulating the proclivity for secondary tumor formation via impairing traditional DNA damage signaling and repair, altering transcriptional changes aimed at maintaining genomic stability following DNA damage, and modulating inflammation within the tumor microenvironment.

## Data Availability

The datasets presented in this study can be found in online repositories. The names of the repository/repositories and accession number(s) can be found below: https://doi.org/doi:10.5061/dryad.0zpc8673k. The supplemental information is being hosted by Zenodo and is now published and publicly available here: https://doi.org/10.5281/zenodo.10641696.

## Supplementary material

SUPPLEMENTARY FIGURE S1 Principal component analysis (PCA) of 12 total RNA samples isolated from WT and KR cells treated with DMSO and etoposide. PCA indicates all replicates of each genotype and treatment combinations, WT-DMSO, WT-etoposide, KR-DMSO, or KR-etoposide, were clustered closely within replicates.

SUPPLEMENTARY FIGURE S2 Differentially expressed genes (DEGs) in DNA-PKcs wild-type (WT) versus kinase-inactive (KR) cells. Each red dot denotes one single gene that has a significant (*p* < 0.05) positive log-fold change, and the blue dot denotes the gene with a significant negative log-fold change. Each black dot defines a gene with a p-value that was not significantly altered (*p* > 0.05). **(A)** DEGs in KR cells compared to WT without etoposide treatment. **(B)** DEGs in KR cells compared to WT after etoposide exposure. **(C)** DEGs in etoposide-exposed WT cells compared to its control counterpart. **(D)** DEGs in etoposide-exposed KR cells compared to its control counterpart.

SUPPLEMENTARY FIGURE S3 Genotype-specific biological pathways altered by differential gene regulation in DNA-PKcs wild-type (WT) versus kinase-inactive (KR) cells without drug exposure. **(A)** Genotype-specific over-represented biological processes populated by upregulated genes in KR compared to WT. **(B)** Genotype-specific under-represented biological processes populated by downregulated genes in KR compared to WT.

SUPPLEMENTARY FIGURE S4 Etoposide-induced biological pathways altered by differential gene regulation in DNA-PKcs wild-type (WT) and kinase-inactive (KR) cells. **(A)** Etoposide-induced upregulated biological processes within WT cells. **(B)** Etoposide-induced downregulated biological processes in WT cells. **(C)** Etoposide-induced upregulated biological processes within KR cells. **(D)** Etoposide-induced downregulated biological processes in KR cells.

SUPPLEMENTARY TABLE S1 Genotypic master regulators controlling the gene expression differences observed in cells producing wild-type (WT) or kinase inactive (KR) DNA-PKcs.

SUPPLEMENTARY TABLE S2 All master regulators controlling the gene expression differences observed in cells producing wild-type (WT) DNA-PKcs following etoposide treatment.

SUPPLEMENTARY TABLE S3 Master regulators controlling the gene expression differences observed in cells producing kinase-inactive (KR) DNA-PKcs following etoposide treatment.

SUPPLEMENTARY TABLE S4 Genotypic master regulators controlling the gene expression differences observed in cells producing wild-type (WT) or kinase inactive (KR) DNA-PKcs after etoposide exposure.
