# Supplementary figures and images for: Comparative analysis of basal and etoposide-induced alterations in gene expression by DNA-PKcs kinase activity
Source: Front Genet. 2024 Mar 21;15:1276365. doi: 10.3389/fgene.2024.1276365 (PMC10991847; doi:10.3389/fgene.2024.1276365)

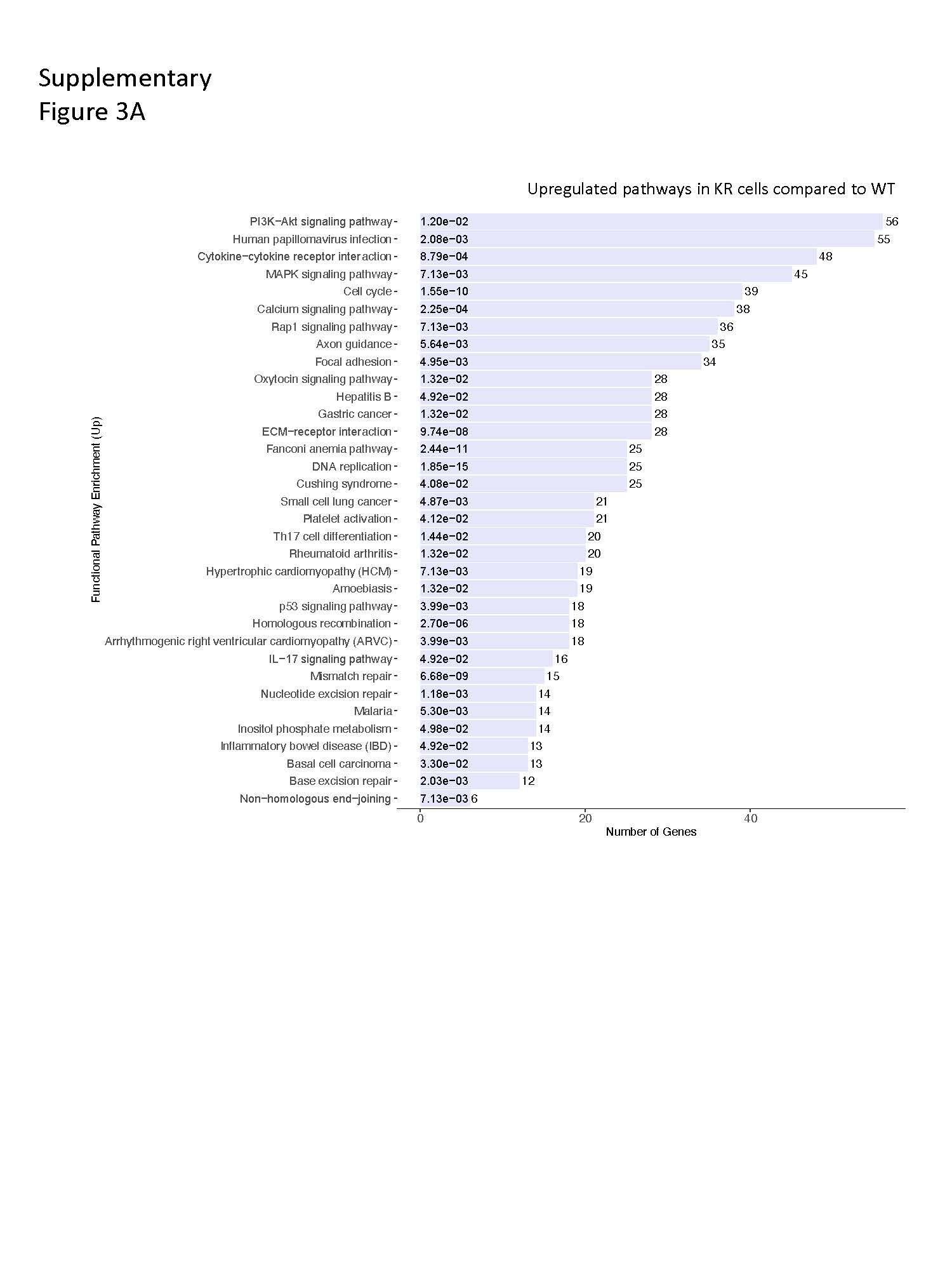

Supplement: Supplementary file 2 [file Image3.JPEG]

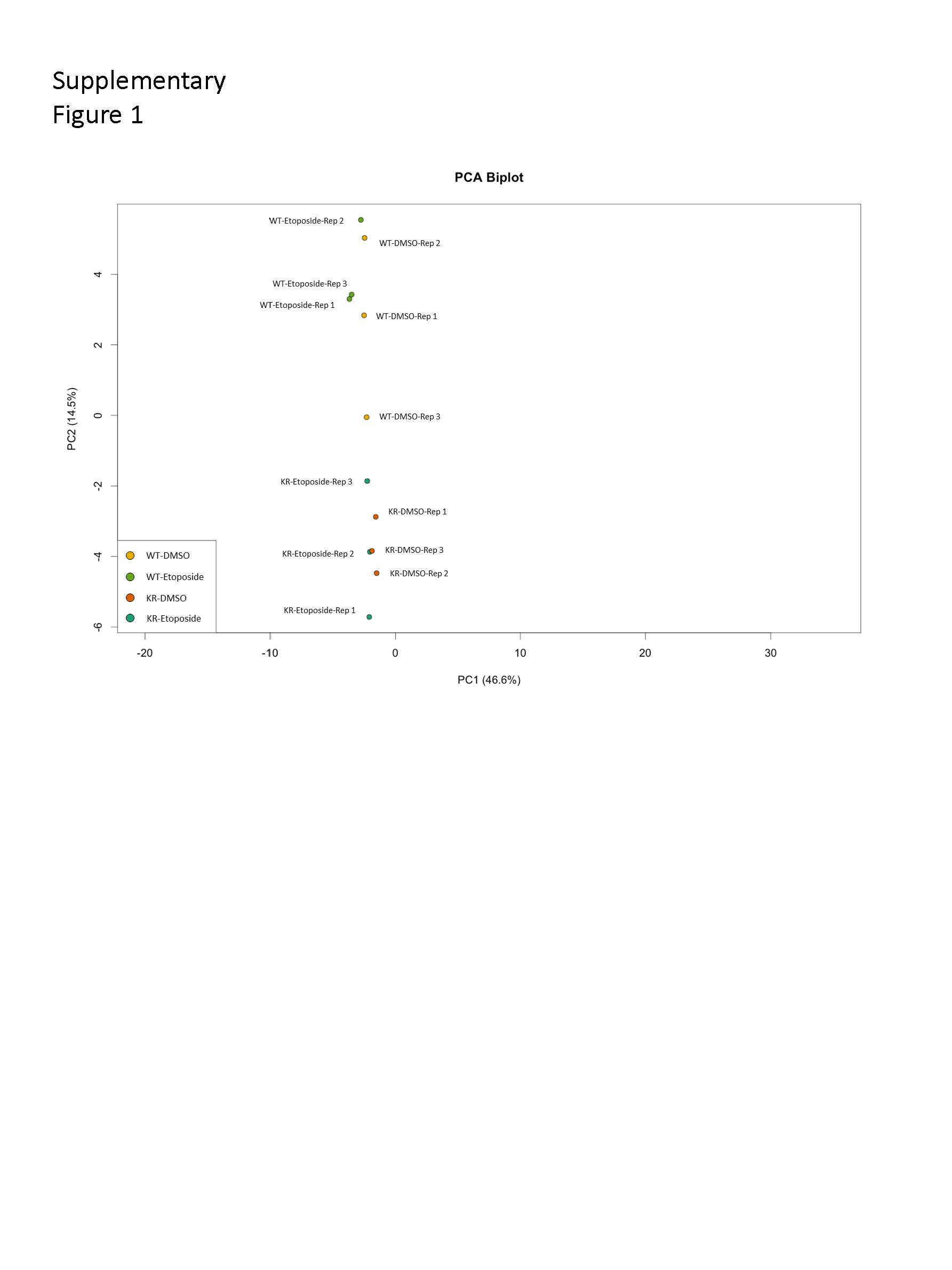

Supplement: Supplementary file 4 [file Image1.JPEG]

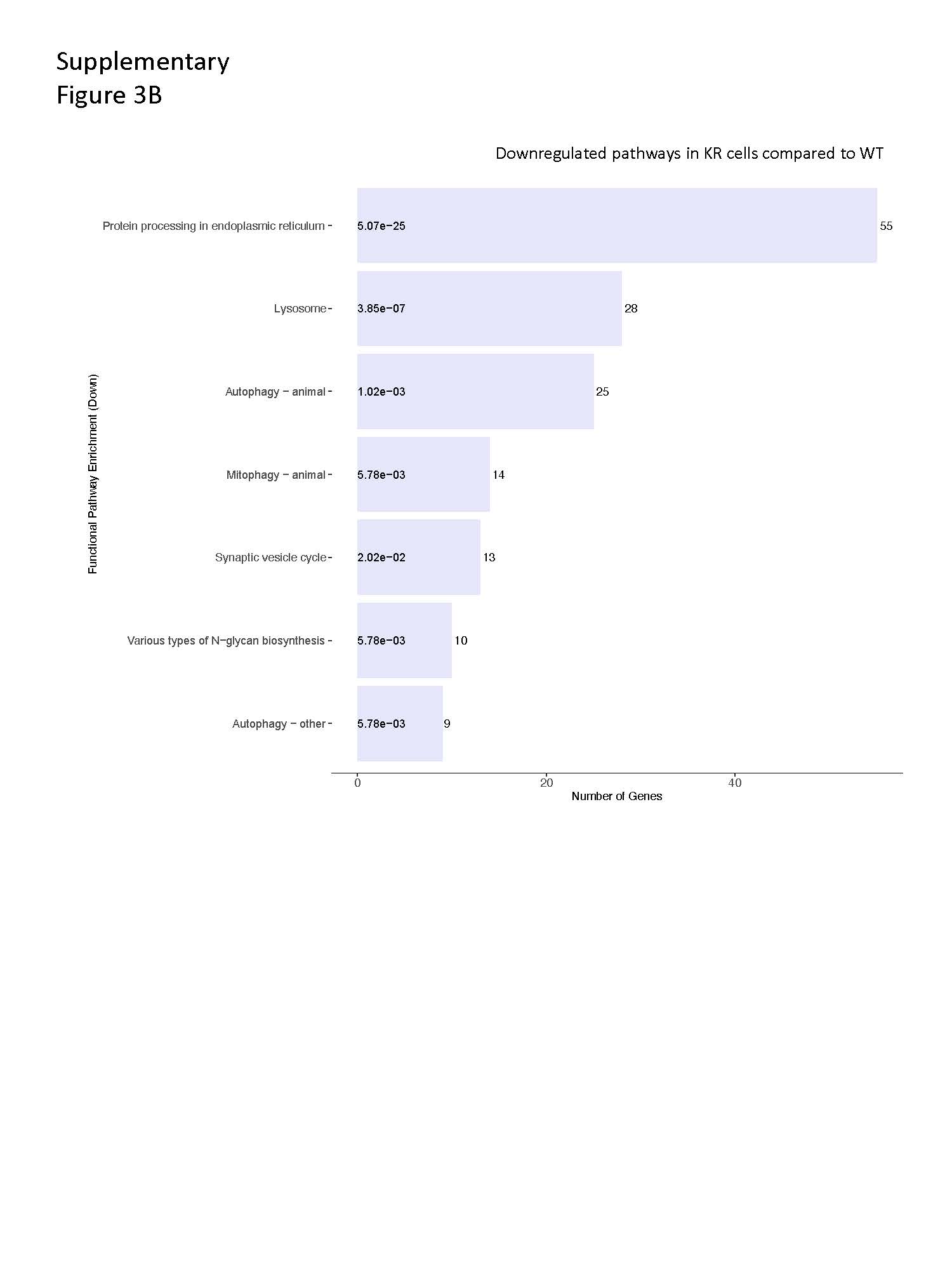

Supplement: Supplementary file 5 [file Image4.JPEG]

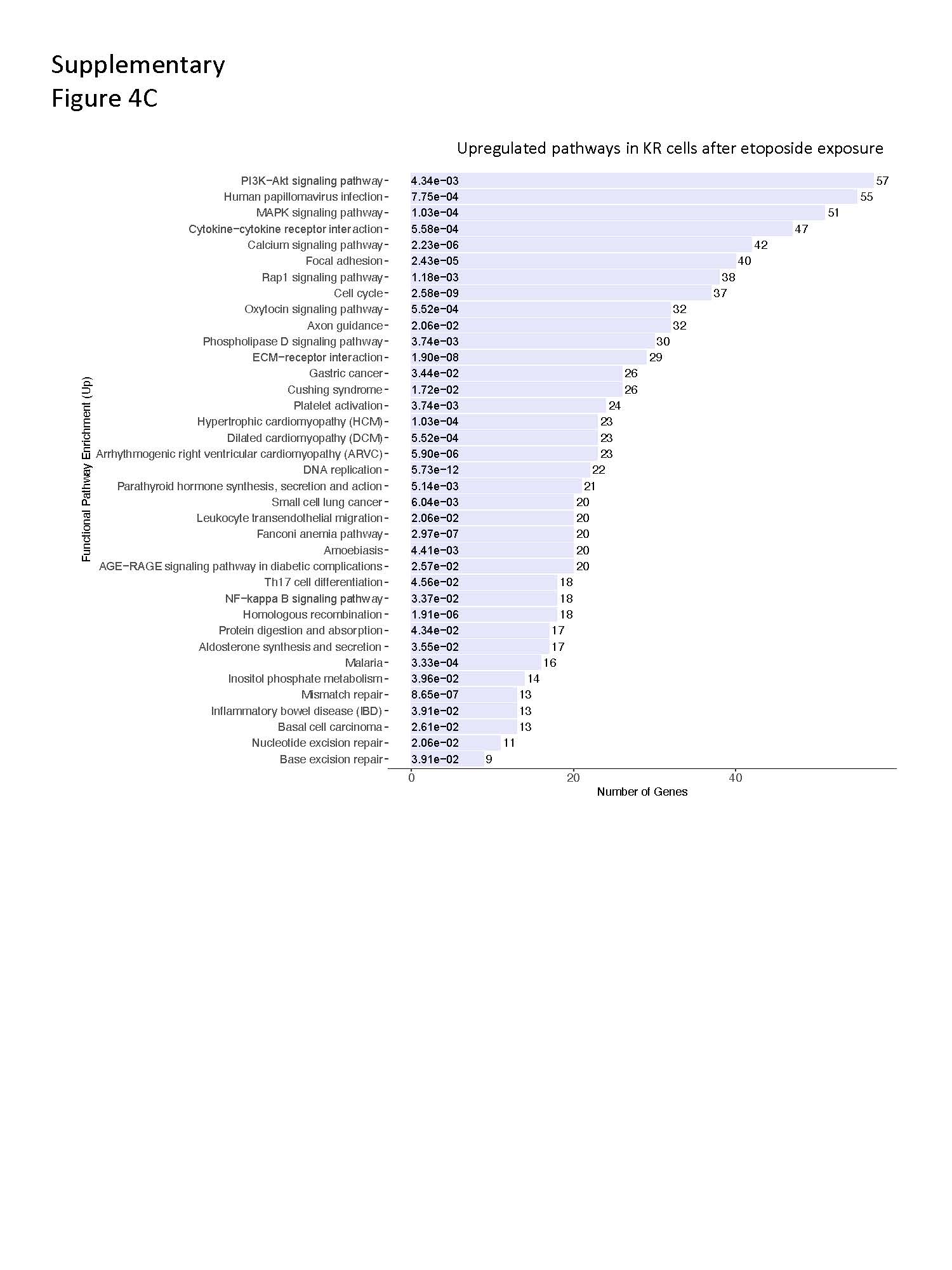

Supplement: Supplementary file 6 [file Image7.JPEG]

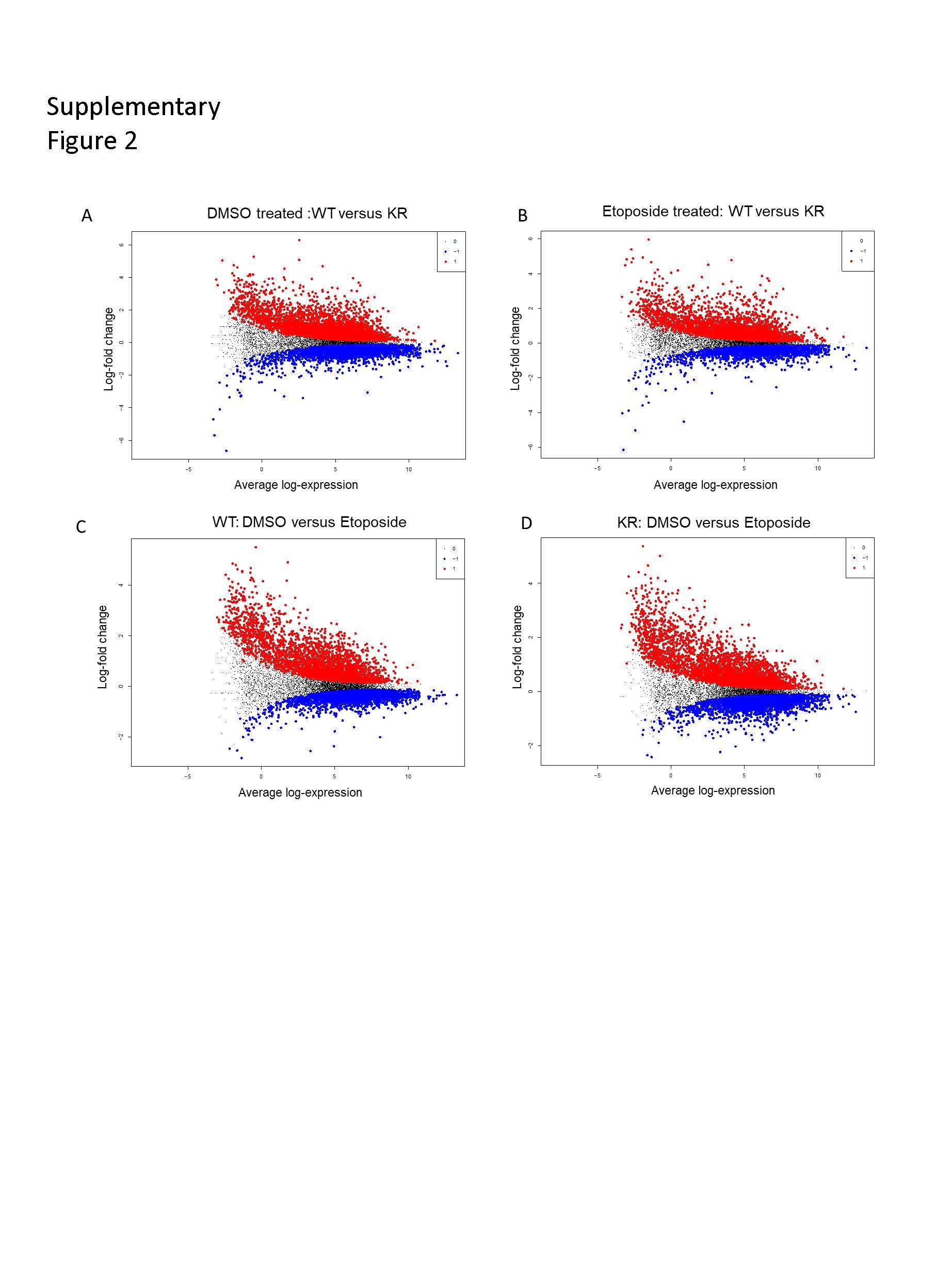

Supplement: Supplementary file 7 [file Image2.JPEG]

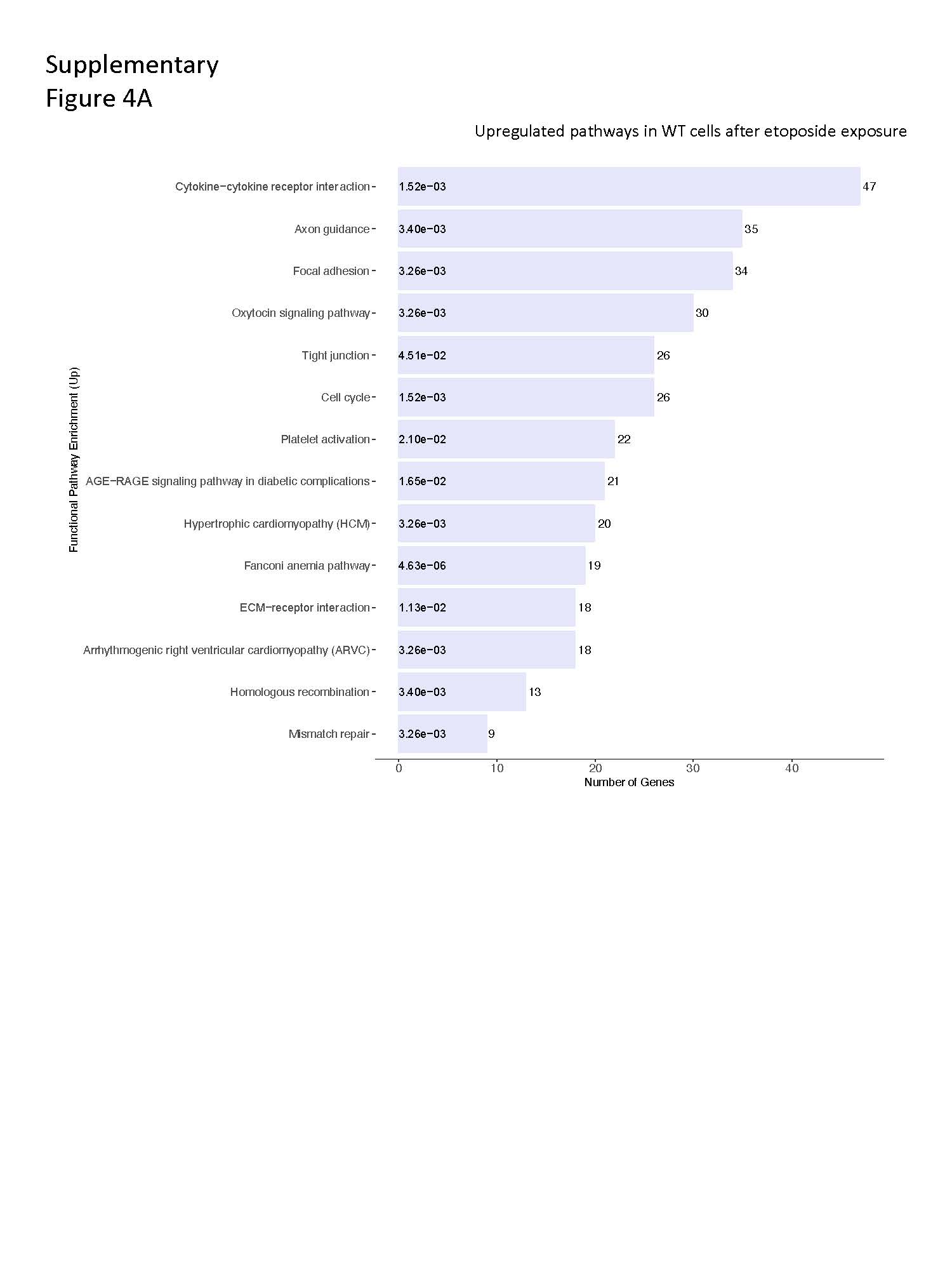

Supplement: Supplementary file 8 [file Image5.JPEG]

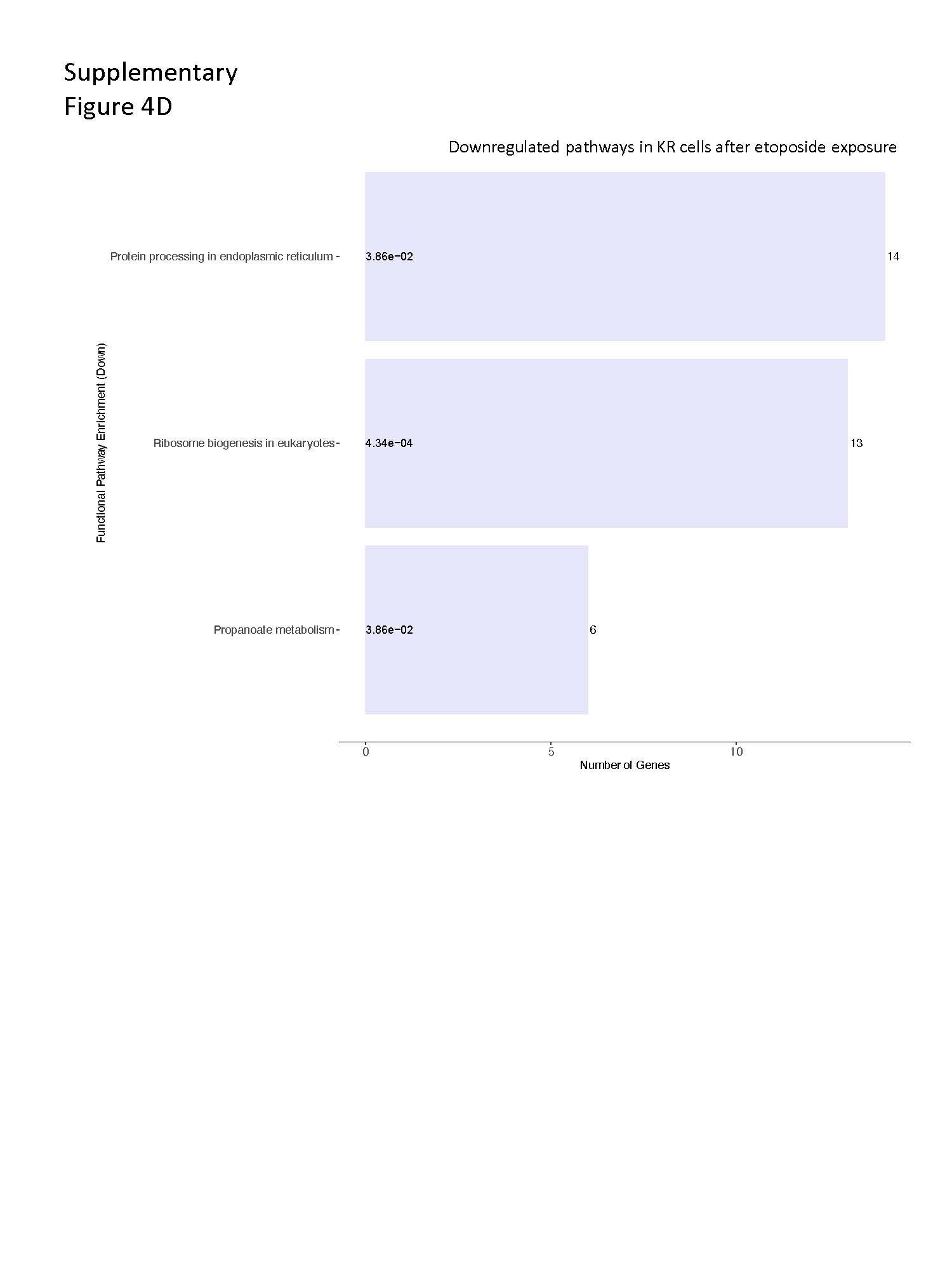

Supplement: Supplementary file 11 [file Image8.JPEG]

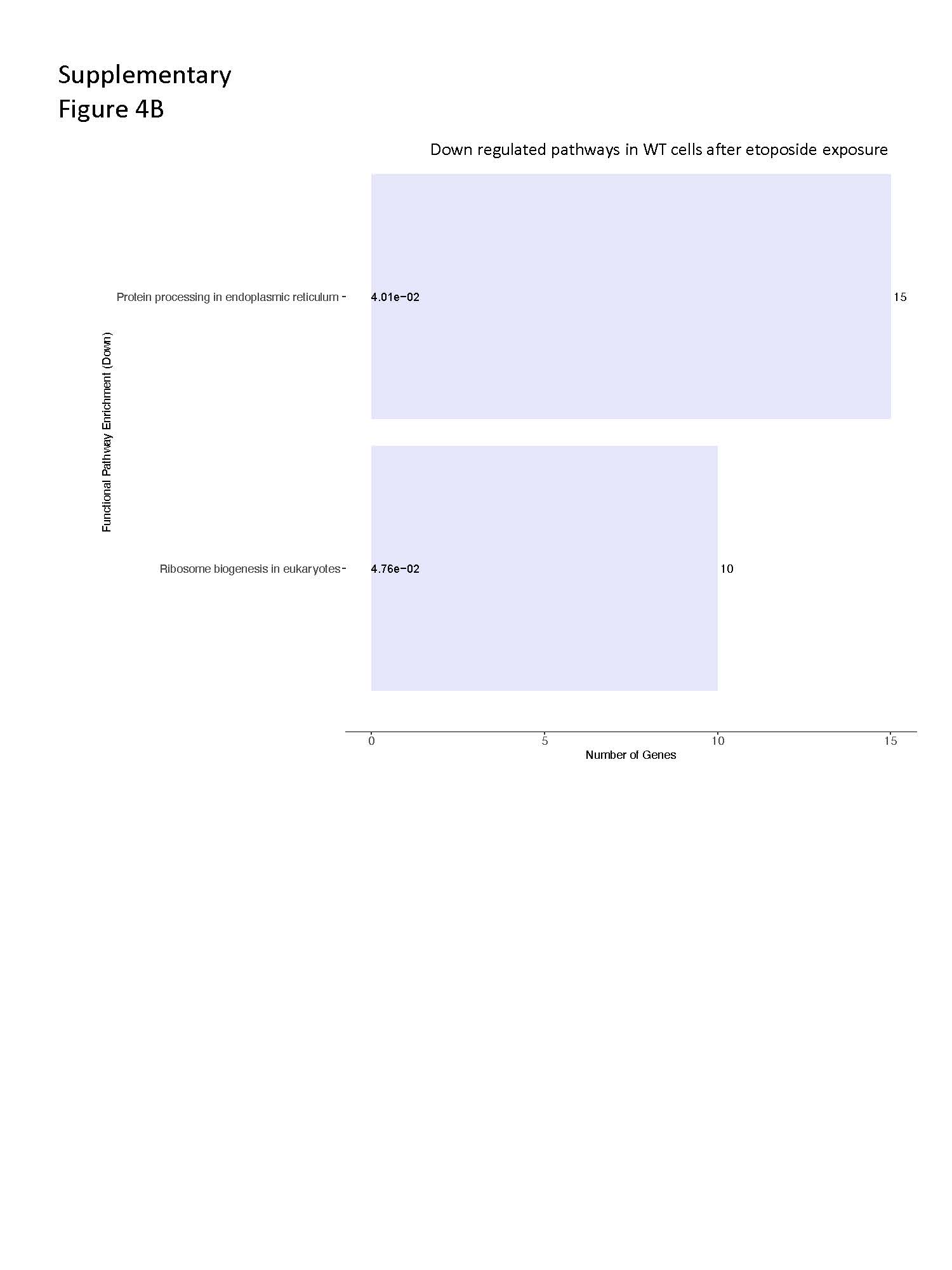

Supplement: Supplementary file 12 [file Image6.JPEG]
